# Comparative Study of Early Versus Late Enteral Feeding in the Postoperative Period Following Small and Large Bowel Surgeries

**DOI:** 10.7759/cureus.79137

**Published:** 2025-02-17

**Authors:** Ajit M Dikle, Saurabh P Dumbre

**Affiliations:** 1 Department of General Surgery, Government Medical College and Hospital, Dharashiv, IND; 2 Department of General Surgery, Postgraduate Institute, Yashwantrao Chavan Memorial (YCM) Hospital, Pimpri Chinchwad Municipal Corporation (PCMC), Pimpri-Chinchwad, IND

**Keywords:** early enteral feeding, ileus duration, postoperative care, small and large bowel surgeries, wound infection rate

## Abstract

Background: The timing of enteral feeding in the postoperative period after small and large bowel surgeries significantly influences patient recovery. Traditional *nil-by-mouth* protocols delay feeding until bowel function returns but may exacerbate malnutrition, delay wound healing, and prolong hospital stays. Emerging evidence suggests early enteral feeding (EEF) offers better outcomes.

Objective: This study aims to compare the effects of EEF (within 24 hours of surgery) and delayed enteral feeding on postoperative recovery, complications, and hospital stay in patients undergoing small and large bowel surgeries.

Methodology: A prospective observational study was conducted at a tertiary rural hospital over two years, including 70 patients undergoing bowel surgeries. Patients were divided into two groups: early feeding (EEF) and late feeding. Outcomes assessed included the duration of ileus, wound infection rates, anastomotic leaks, feeding tolerance, and length of hospital stay. Statistical analysis was performed using chi-square tests and t-tests, with *P* < 0.05 considered significant.

Results: The mean duration of ileus was significantly shorter in the EEF group (4.23 ± 1.26 days) than in the late feeding group (5.6 ± 1.4 days; *P* < 0.01). The EEF group also had a shorter hospital stay (5.74 ± 1.44 days vs. 7.11 ± 1.23 days; *P* < 0.01). Complications, including wound infections (2.9% vs. 11.4%) and anastomotic leaks (0% vs. 8.6%), were lower in the EEF group. Feeding tolerance was comparable between the groups, with 94.3% of patients with EEF tolerating feeding well.

Conclusions: EEF significantly reduces ileus duration, hospital stay, and complications, demonstrating its safety and effectiveness. These findings support incorporating EEF into standard postoperative care protocols to improve outcomes and reduce healthcare costs. Further multicentric studies are recommended to validate these results across diverse settings.

## Introduction

Postoperative care plays a crucial role in determining the recovery trajectory of patients undergoing gastrointestinal surgeries. One common practice is the period of starvation, or *nil by mouth*, which is traditionally implemented to allow the gastrointestinal tract to recover and prevent complications such as anastomotic leaks or nausea. However, this practice has been questioned due to its potential to exacerbate malnutrition, delay wound healing, and prolong hospital stays [1,2].

The gastrointestinal tract demonstrates varying recovery patterns following surgery, with the small bowel regaining function within four to eight hours, while the stomach and colon may take up to 48 hours and three to five days, respectively. Despite these recovery timelines, patients are often subjected to prolonged fasting, leading to adverse effects like compromised immune function, delayed anastomotic healing, and increased susceptibility to infections. These challenges necessitate reevaluating the timing of enteral feeding in the postoperative period [3,4].

Emerging evidence suggests that early enteral feeding (EEF) within 24 hours of surgery offers significant advantages. EEF has been associated with enhanced wound healing, reduced rates of infectious complications, and shorter hospital stays [5]. It also helps modulate inflammatory and immune responses while providing critical nutritional support at a time when the body requires it the most. Early feeding has shown promise in minimizing the duration of postoperative ileus, improving patient tolerance, and reducing the incidence of complications like anastomotic leaks [1,6,7].

In contrast, delayed feeding, often initiated days after surgery, continues to be a widely practiced approach due to concerns about potential stress on surgical repairs and complications like nausea or vomiting [8]. However, studies have demonstrated that gastrointestinal secretions present at the anastomotic site are not significantly affected by early feeding, and prolonged fasting offers no additional benefit in terms of wound healing [9,10].

This study aims to compare the outcomes of EEF versus late enteral feeding in patients undergoing small and large bowel surgeries [11]. The objectives include evaluating the incidence of anastomotic leaks, the onset and duration of paralytic ileus, wound infection rates, tolerance to enteral feeding, and the length of hospital stay. By addressing these parameters, the study seeks to contribute to the growing body of evidence supporting optimized postoperative nutritional strategies, ultimately improving patient recovery and reducing healthcare costs.

## Materials and methods

This prospective observational study was carried out in the Department of General Surgery at Maharashtra Institute of Medical Education and Research (MIMER) Medical College, Talegaon Dabhade, India, a tertiary rural hospital, over two years from January 2021 to December 2022. The study aimed to evaluate the effects of EEF versus late enteral feeding on postoperative outcomes in patients undergoing small and large bowel surgeries. Ethical approval was obtained from the institutional ethics committee, and informed consent was secured from all participants before enrollment. The study population included patients undergoing various bowel surgeries, such as resection and anastomosis, ileostomy or colostomy closures, and surgeries for diverticula or bowel perforations. Eligibility criteria were strictly adhered to, including adult patients aged 18 years and above who were candidates for elective or emergency bowel surgeries. Exclusion criteria ensured the exclusion of vulnerable populations such as patients under 18 years, pregnant women, and immunocompromised individuals, including those with HIV or on immunosuppressive therapy. Other exclusion parameters included gross intra-abdominal contamination, postoperative stomas, sustained bowel ischemia, and patients classified as ASA (American Society of Anesthesiologists) grade 4 or higher. Laparoscopic procedures and cases requiring postoperative ventilator support were also excluded.

A total of 70 patients were included in the study, with 35 patients allocated to each of the two study groups. The allocation was based on the timing of their enteral feeding post-surgery. The early feeding group consisted of patients who were initiated on enteral feeding within 24 hours postoperatively. This group began with sips of water administered through a Ryle’s tube, which was progressively advanced to polymeric feeds as tolerated. The Ryle’s tube was removed once patients demonstrated adequate tolerance to oral feeding. In contrast, the late feeding group was kept nil by mouth until the return of bowel function, as indicated by the passage of flatus and the presence of active bowel sounds. Feeding in this group was introduced only after these signs were observed. The nutritional regimen for all patients followed a standardized protocol guided by the hospital’s nutritionist. The polymeric feeds were initiated at a rate of 100 mL/hour on the first postoperative day and were increased to 200 mL/hour on the second day, contingent on the patient’s tolerance. The progression of feeding was closely monitored, and adjustments were made based on individual responses.

The study assessed several postoperative outcomes to compare the two groups. These included the incidence of anastomotic leaks, onset and duration of paralytic ileus, wound infection rates, tolerance to enteral feeding, and length of hospital stay. Data were meticulously recorded in a structured Proforma, capturing details such as drain output, gastrointestinal symptoms, and any signs of infective or surgical complications. Data analysis was performed using IBM SPSS Statistics 26.0 (IBM Corp., Armonk, NY). Qualitative variables, such as the incidence of complications, were expressed as frequencies and percentages. Quantitative variables, including the duration of ileus and hospital stay, were represented as mean ± standard deviation. Statistical comparisons between the two groups were conducted using the chi-square test for qualitative data and the unpaired t-test or Mann-Whitney U test for quantitative data, depending on the normality of the data distribution. A *P*-value of less than 0.05 was considered statistically significant.

## Results

The study included 70 patients, with a mean age of 44.87 ± 6.7 years. Among the participants, 14 (20%) were aged 40 years or younger, 44 (62.9%) were between 41 and 50 years, and 12 (17.1%) were aged 51-60 years. The gender distribution comprised 43 (61.4%) males and 27 (38.6%) females. The most common indication for surgery was colostomy closure (30, 42.9%), followed by hemicolectomy (17, 24.3%), small bowel resection (12, 17.1%), proctocolectomy (6, 8.6%), and pyloric perforation (5, 7.1%) (Table 1).

**Table 1:** Distribution of study subjects according to age (years), gender, and induction of surgery.

Parameter		Frequency	Percentage
Age (years)	<=40	14	20.0%
	41-50	44	62.9%
	51-60	12	17.1%
Gender	Male	27	38.6%
	Female	43	61.4%
Indication of surgery	Colostomy closure	30	42.9%
	Hemicolectomy	17	24.3%
	Proctocolectomy	6	8.6%
	Pyloric perforation	5	7.1%
	Small bowel resection	12	17.1%

When comparing the mean age between the early and late feeding groups, no significant difference was observed, with mean ages of 45.09 ± 6.35 years and 44.66 ± 7.18 years, respectively (*P* = 0.792, Table 2). Gender distribution also showed no statistically significant difference between the groups. In the early feeding group, 16 (45.7%) were females and 19 (54.3%) were males, while in the late feeding group, 11 (31.4%) were females and 24 (68.6%) were males (Table 3).

**Table 2:** Comparison of the mean age in early and late start of feeding. SD, standard deviation

	Start of feeding	n	Mean	SD	*t*-stat	*P*-value
Age (years)	Early	35	45.09	6.35	0.27	0.792
	Late	35	44.66	7.18		
	Total	70	44.87	6.7		

**Table 3:** Cross-tabulation of gender and timing of feeding.

	Early, *n* (%)	Late, *n* (%)	Total, *n* (%)
Female	16 (45.7%)	11 (31.4%)	27 (38.6%)
Male	19 (54.3%)	24 (68.6%)	43 (61.4%)
Total	35 (100%)	35 (100%)	70 (100%)

The study evaluated the association between the timing of feeding and various outcomes. Tolerance to feeding was excellent in both groups, with 33 (94.3%) in the early group and 34 (97.1%) in the late group reporting no issues (*P* = 0.555; Table 4). Regarding surgical indications, the distribution of patients across different procedures was comparable between the groups, with no significant difference (*P* = 0.628; Table 4).

**Table 4:** Association of timing of the start of feeding with an indication for surgery, tolerance to feeding, and complications.

Parameter		Early	Late	Total	Chi-square	*P*-value
Indication of surgery	Colostomy closure	18 (51.4%)	12 (34.3%)	30 (42.9%)	2.596	0.628
	Hemicolectomy	7 (20.0%)	10 (28.6%)	17 (24.3%)		
	Proctocolectomy	2 (5.7%)	4 (11.4%)	6 (8.6%)		
	Pyloric perforation	2 (5.7%)	3 (8.6%)	5 (7.1%)		
	Small bowel resection	6 (17.1%)	6 (17.1%)	12 (17.1%)		
Tolerance to feeding	No	2 (5.7%)	1 (2.9%)	3 (4.3%)	0.348	0.555
	Yes	33 (94.3%)	34 (97.1%)	67 (95.7%)		
Complications	Gastrointestinal	2 (5.70%)	5 (14.30%)	7 (10.0%)	-1.21	0.227
	Anastomotic leak	0 (0.0%)	3 (8.60%)	3 (4.30%)	-1.81	0.070
	Wound Infections	1 (2.90%)	4 (11.40%)	5 (7.10%)	-1.41	0.158

The duration of postoperative ileus was significantly shorter in the early feeding group, averaging 4.23 ± 1.26 days compared to 5.6 ± 1.4 days in the late feeding group (*P *< 0.01; Table 5). Complications such as gastrointestinal issues, anastomotic leaks, and wound infections were lower in the early feeding group. Gastrointestinal complications occurred in 2 (5.7%) of the early group and 5 (14.3%) of the late group (*P *= 0.227). Anastomotic leaks were observed only in the late feeding group (6, 8.6%), with none reported in the early group (*P* = 0.070). Wound infections were also higher in the late feeding group (11.4% vs. 2.9%, *P* = 0.158; Table 5).

**Table 5:** Comparison of duration of ileus (days) in early and late start of feeding. SD, standard deviation

Variables	Start of feeding	n	Mean	SD	*t*-stat	*P*-value
Duration of ileus (Days)	Early	35	4.23	1.26	-4.30	<0.01
	Late	35	5.6	1.4		

These findings highlight the benefits of EEF in reducing the duration of ileus and minimizing postoperative complications, contributing to improved outcomes for patients undergoing bowel surgeries.

## Discussion

Postoperative management, particularly the timing of enteral feeding, plays a critical role in determining patient outcomes after bowel surgeries. Traditionally, delayed feeding has been practiced to mitigate potential risks such as anastomotic stress, nausea, and vomiting. However, this study, in alignment with emerging evidence, demonstrates the significant advantages of EEF over delayed feeding in terms of reducing complications, accelerating recovery, and improving overall outcomes.

In this study, the mean duration of ileus was significantly shorter in the EEF group (4.23 ± 1.26 days) compared to the late feeding group (5.6 ± 1.4 days). This finding aligns with studies by Nematihonar et al. [8] and Dag et al. [9], who also observed faster resolution of ileus in patients receiving early feeding. The shorter duration of ileus in the EEF group may be attributed to the stimulation of gut motility by luminal nutrition, which prevents morphological and functional alterations associated with prolonged fasting. Physiologically, bile acids play a crucial role in stimulating intestinal motility by promoting water and electrolyte secretion, preventing luminal stasis and bacterial overgrowth. Moreover, bile acids interact with the enteric nervous system and modulate hormone release, including cholecystokinin (CCK) and motilin, which promote coordinated intestinal contractions, further aiding in ileus resolution [10].

Hospital stay was another parameter significantly impacted by the timing of feeding. Patients in the EEF group had a shorter average stay (5.74 ± 1.44 days) compared to the late feeding group (7.11 ± 1.23 days). Similar results were reported in studies by Nematihonar et al. [8] and Herbert et al. [11], who demonstrated that early feeding reduces hospitalization by minimizing complications and promoting faster recovery. The shorter hospital stay in the EEF group translates into lower healthcare costs and better resource utilization. This improvement may be linked to the ability of EEF to maintain gut integrity by stimulating peristalsis and reducing bacterial translocation while also enhancing enteric nervous system activity to accelerate ileus resolution [12].

The incidence of complications, including wound infections, anastomotic leaks, and gastrointestinal issues, was lower in the EEF group. Wound infections occurred in 2.9% of the EEF group compared to 11.4% in the late feeding group. No anastomotic leaks were observed in the EEF group, whereas the late feeding group reported an 8.6% rate. These results are consistent with studies by Deng et al. [13] and Zhuang et al. [14], who noted that early feeding improves wound healing and reduces infectious complications. The improved outcomes with EEF can be attributed to its role in modulating immune and inflammatory responses and maintaining gut integrity, as early enteral nutrition prevents mucosal atrophy and reduces bacterial translocation, ultimately leading to fewer infections and faster healing [15].

Tolerance to feeding was high in both groups, with no statistically significant difference. In the EEF group, 94.3% of patients tolerated feeding well, compared to 97.1% in the late feeding group. This indicates that early initiation of feeding is feasible and does not pose additional risks to most patients. These findings are supported by studies like those of Reissman et al. [16], who concluded that early feeding is safe and well-tolerated.

## Conclusions

Despite the promising results, there were limitations in this study, including a relatively small sample size and its single-center design. While the findings are consistent with existing literature, larger, multicentric studies are required to confirm the benefits of EEF across diverse patient populations and surgical contexts. In conclusion, EEF after bowel surgeries is associated with significant clinical benefits, including a shorter duration of ileus, reduced hospital stays, and lower rates of complications such as wound infections and anastomotic leaks. The study highlights the safety, feasibility, and effectiveness of EEF as a postoperative nutritional strategy, paving the way for its broader adoption in clinical practice.
